# Surgery or comfort care for neonates with surgical necrotizing enterocolitis: Lessons learned from behavioral artificial intelligence technology

**DOI:** 10.3389/fped.2023.1122188

**Published:** 2023-02-28

**Authors:** Otis C. van Varsseveld, Annebel ten Broeke, Caspar G. Chorus, Nicolaas Heyning, Elisabeth M. W. Kooi, Jan B. F. Hulscher

**Affiliations:** ^1^ Department of Surgery, Division of Pediatric Surgery, University Medical Center Groningen, University of Groningen, Groningen, Netherlands; ^2^ Councyl, Delft, Netherlands; ^3^ Department of Engineering Systems and Services, Faculty Technology Policy and Management, Delft University of Technology, Delft, Netherlands; ^4^ Department of Neonatology, Beatrix Children’s Hospital, University Medical Center Groningen, University of Groningen, Groningen, Netherlands

**Keywords:** necrotizing enterocolitis, decision making, artificial intelligence, choice analysis, critical care, comfort care, decision support

## Abstract

**Background:**

Critical decision making in surgical necrotizing enterocolitis (NEC) is highly complex and hard to capture in decision rules due to case-specificity and high mortality risk. In this choice experiment, we aimed to identify the implicit weight of decision factors towards future decision support, and to assess potential differences between specialties or centers.

**Methods:**

Thirty-five hypothetical surgical NEC scenarios with different factor levels were evaluated by neonatal care experts of all Dutch neonatal care centers in an online environment, where a recommendation for surgery or comfort care was requested. We conducted choice analysis by constructing a binary logistic regression model according to behavioral artificial intelligence technology (BAIT).

**Results:**

Out of 109 invited neonatal care experts, 62 (57%) participated, including 45 neonatologists, 16 pediatric surgeons and one neonatology physician assistant. *Cerebral ultrasound* (Relative importance = 20%, OR = 4.06, 95% CI = 3.39–4.86) was the most important factor in the decision surgery versus comfort care in surgical NEC, nationwide and for all specialties and centers. Pediatric surgeons more often recommended surgery compared to neonatologists (62% vs. 57%, *p* = 0.03). For all centers, *cerebral ultrasound*, *congenital comorbidity*, *hemodynamics* and *parental preferences* were significant decision factors (*p* < 0.05). *Sex* (*p* = 0.14), *growth since birth* (*p* = 0.25), and *estimated parental capacities* (*p* = 0.06) had no significance in nationwide nor subgroup analyses.

**Conclusion:**

We demonstrated how BAIT can analyze the implicit weight of factors in the complex and critical decision for surgery or comfort care for (surgical) NEC. The findings reflect Dutch expertise, but the technique can be expanded internationally. After validation, our choice model/BAIT may function as decision aid.

## Introduction

While artificial intelligence as decision support is rapidly gaining ground in medicine (1–5), the use of artificial intelligence in the context of moral decisions is much less developed (6). Traditional rule-based decision support systems fail to capture the complexity and subtlety involved in medical decision making (2). Recently, we presented Behavioral Artificial Intelligence Technology (BAIT) as a novel approach to digitally capture expertise. The BAIT approach is a reconceptualization of econometric techniques, namely conjoint analysis and discrete choice theory, to generate decision transparency and support for medical experts (7). We have used BAIT in two single center pilot studies in the context of both an adult intensive care setting regarding COVID-19, and a neonatal intensive care setting regarding necrotizing enterocolitis (NEC) (7, 8). In both pilot studies BAIT provided insight into implicit decision trade-offs. In the present paper we will illustrate the use of this technique in cases of NEC on a nationwide and multicenter scale, as it may function as an important future adjunct in moral medical decision making.

NEC is a dreadful disease of the neonatal intestines, with an incidence varying between 3% and 17% in very low birth weight neonates (<1,500 g) (9–13). NEC incidence is increasing due to generally improved survival of the most preterm infants (14). Despite advances in neonatal care, mortality rates of NEC may still reach up to 40% (14, 15). For approximately one in three neonates with NEC, emergency laparotomy is necessary within hours to days after onset when conservative management does not suffice (surgical NEC) (16). However, perioperative mortality can reach 50%, and long-term morbidity, such as gastrointestinal complications and neurodevelopmental delay, occurs in over 75% (17). Hence, each case in which surgery becomes necessary poses both the treating medical team and the parents with the urgent dilemma whether surgery is still in the child’s best interest (10, 18).

The aim of the current study is to identify, interpret and further elucidate the implicit weights of decision factors in a national context and to identify possible between-group variations that contribute to critical decision making, in the context of one of the most difficult decisions in medicine: surgery versus comfort care for a critically ill preterm neonate with surgical NEC (18, 19). This may offer future decision support and educational insights to evaluate decision making and improve collaboration between stakeholders. Towards this goal, we assessed decision making in surgical NEC nationwide (the Netherlands), and subsequently focused on the differences between neonatologists and pediatric surgeons, between neonatal centers and between more and less experienced physicians, using the BAIT technology.

## Methods

In a previous pilot study, we have developed a decision-analysis tool for NEC based on BAIT (7), which was employed on a larger scale for the current study. The BAIT technique comprises four steps: (1) definition of the expert decision and relevant factors; (2) determination of choice model structure; (3) design and execution of the choice experiment; (4) results analysis. The study was approved by the University Medical Center Groningen (UMCG) Ethical Board (METc 2020/310) and all methods were carried out in accordance with relevant guidelines and regulations.

### Definition of the expert decision and relevant factors

First, an expert group of two senior neonatologists and two senior pediatric surgeons, defined the medical decision as follows: “to advise parents to proceed to surgery or to initiate comfort care (palliative care, resulting in death) for a critically ill infant with confirmed NEC and clear indication for surgery. This indication is a given fact for the sake of this experiment and is in daily practice always discussed within the multidisciplinary team treating the child (neonatologist, pediatric surgeon and pediatric anesthetist), and can consist of intestinal perforation confirmed by abdominal imaging and/or clinical deterioration despite maximum active conservative treatment”. This description captures the actual clinical situation as closely as possible.

The same expert group subsequently identified fourteen presumably relevant factors in the decision and their ranges (Table 1), also using the data from our pilot study. Certain factor levels were purposely formulated in a subjective fashion rather than based on instrumental measurements (e.g., cerebral ultrasound “good prognosis” rather than “no intraventricular hemorrhage”). This served two goals: (1) experts usually form a personal conclusion (good/intermediate/weak) for certain decision factors based on multiple clinical/objective inputs, so this resembles the clinical situation as closely as possible and; (2) we specifically aimed to capture subjective morality in our study. Constraining factor combinations in real life were specified for exclusion from the scenarios presented in the choice experiment as they will not occur in real-life cases. Excluded combinations were: (1) a *gestational age* of 24 or 26 weeks with a *birth weight* of 1,500 g; (2) *gestational age* of 30 weeks with a *birth weight* of 500 or 650 g; (3) no complications since birth in combination with poor lung function and/or poor neurodevelopmental prognosis from *cerebral ultrasound*.

**Table 1:** Predetermined decision factors and their ranges within the predefined levels.

Factor	Level 1	Level 2	Level 3	Level 4
Sex	Boy	Girl		
Gestational age	24 weeks	26 weeks	28 weeks	30 weeks
Birth weight	500 grams	650 grams	800 grams	1,500 grams
Perinatal asphyxia	Yes	Dubious	No	
Congenital comorbidity	Present with high impact	Present with minor impact	Absent	
Clinical course pre-NEC	Serious complications	Minor complications	No complications	
Postnatal age	0–7 days	7–14 days	14–21 days	
Growth since birth	Weak	Intermediate	Good	
Cerebral ultrasound	Poor prognosis	Intermediate prognosis	Good prognosis	
Lung function	Weak	Intermediate	Good	
Hemodynamics	Unstable despite maximum inotropic support	Stable with inotropic support	Stable without inotropic support	
Cerebral oxygenation (NIRS rSO_2_)	40	60	80	
Parental preferences	In favor of comfort care	Doubtful about surgery	In favor of surgery	
Estimated parental capacities (for future care for NEC survivor)	Weak	Intermediate	Good	

NEC, necrotizing enterocolitis; NIRS, near-infrared spectroscopy.

### Determination of choice model structure

Second, the choice model structure was defined. We opted for a binary logistic regression model, because of the positive and intuitive results achieved in our pilot study (7). For transparency and interpretability, the weight of factors was modelled linearly (e.g., a positive linear impact of increasing *gestational age* towards the decision to operate).

### Design and execution of the choice experiment

**Figure 1:**
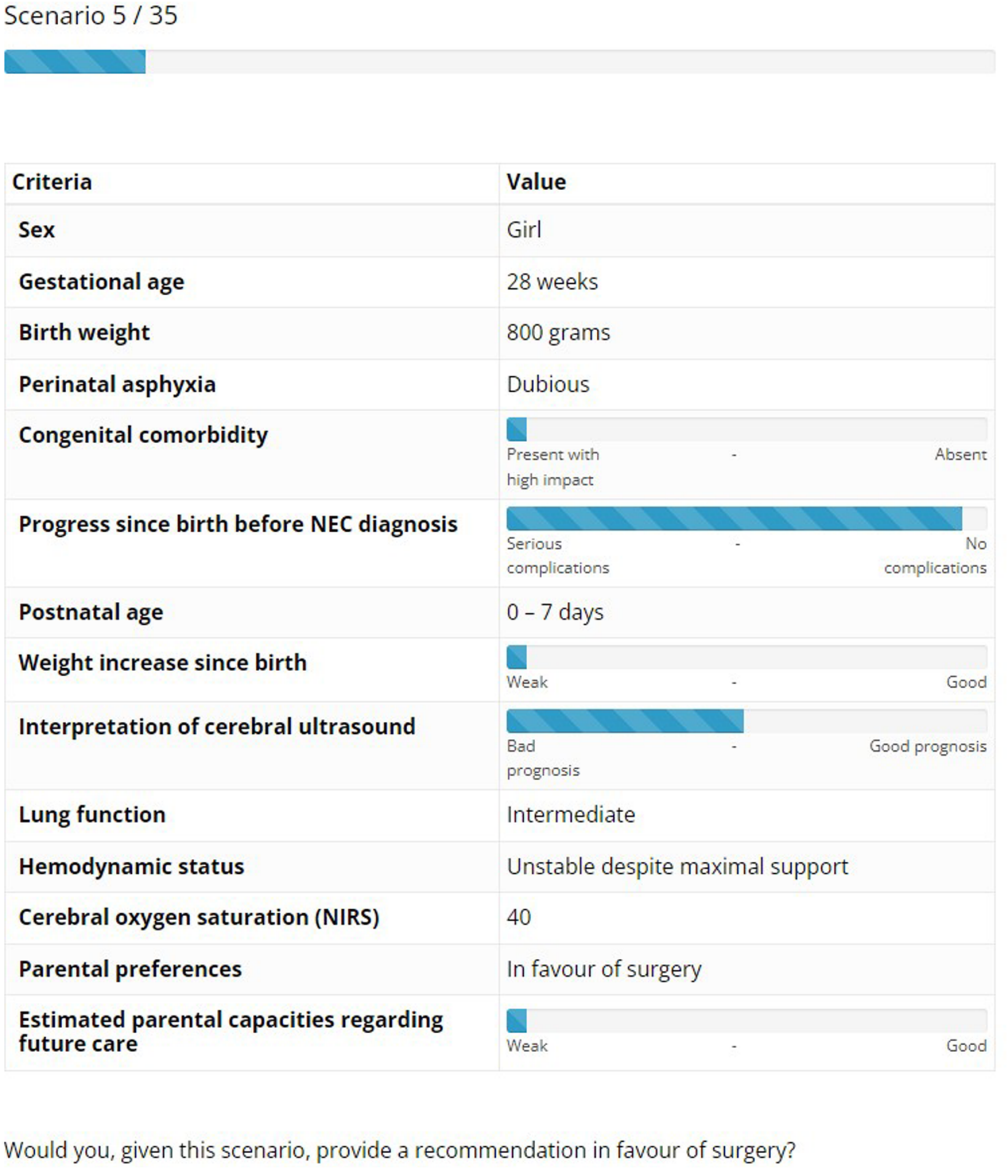
Example of a choice scenario as completed by participants.

Third, the choice experiment was designed and executed. This consisted of scenarios mimicking real-life cases of surgical NEC patients, where a hypothetical yes/no choice should be made by the participating expert based on the provided factors (Figure 1). Efficient design techniques by Ngene software (version 1.2.1, ChoiceMetrics) ensured that the maximum amount of information regarding factor weights was obtained with each completed scenario. This entailed that a total of 35 scenarios were created for each participant. For each participant, the first two scenarios were extremes (i.e., maximum positive and negative values), functioning as a form of positive and negative control for general NEC expertise. Subsequent scenario order was randomized for each participant.

All neonatologists, pediatric surgeons and physician assistants of all tertiary neonatal care centers in the Netherlands were invited to participate, to allow representation of all Dutch care providers with expertise and involvement in NEC care. Participants were invited through e-mail to complete the experiment in an online application (WEM No-Code Platform).

### Statistical analysis

**Figure 2:**
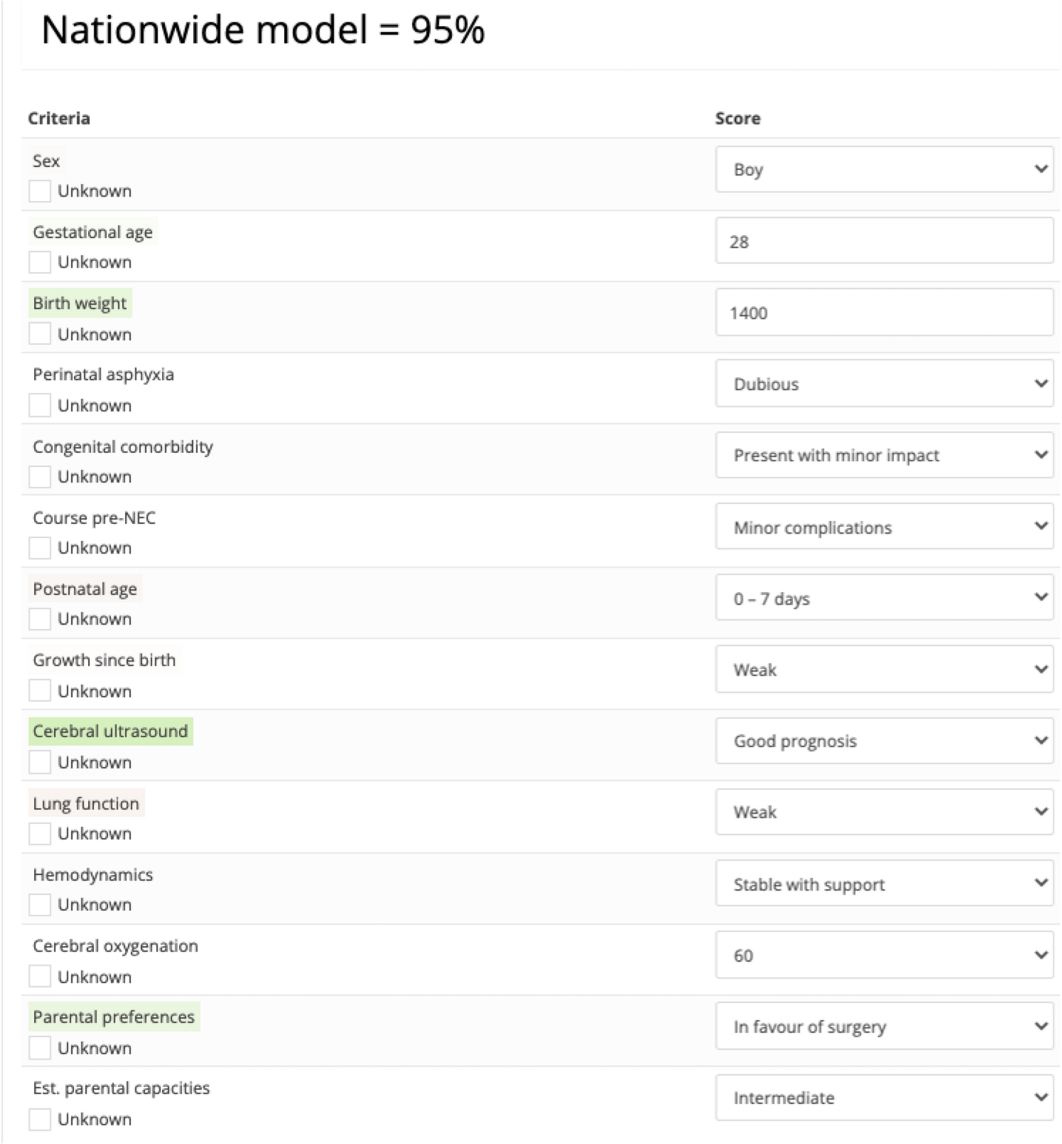
Example of an assessment generated by the nationwide choice model stating that the probability that a randomly sampled expert from the expert group would recommend to perform surgery on a patient with this profile equals 95%. Color coding highlights which factors had a positive or negative contribution to the assessment.

The fourth step was analysis of choices that were observed in the choice experiment. For analysis we utilized Apollo (version 0.2.4, package in R) for logistic regression to estimate the importance weight of each factor, including their signs (positive or negative), with the maximum likelihood technique. We provide the logistic regression beta coefficient and odds ratio (OR) of each factor and its significance in regression analysis (*p*-value of factor in estimated model vs. null model). A two-tailed *p*-value <0.05 was considered significant. The attained choice model equipped with the estimated weights was subsequently used to assess particular hypothetical choice situations, including cases not presented in the actual choice experiment. By combining the estimated effect of different decision factors, the model forms a probability statement (percentage) that an expert that is randomly sampled from the expert group would advise to perform surgery on a patient with the given profile (Figure 2). Model fit is expressed as McFadden’s *ρ*^2^, calculated by the ratio of the maximized log likelihood (predictive model) and the null log likelihood (null model). Values between 0.2 and 0.4 indicate a good model fit (20), particularly for the type of choices made in experimental conditions with difficult trade-offs.

The relative importance (RI) was determined as follows: for each factor, the estimated weight is multiplied by the range of the factor (theoretical maximum effect of the factor). RI is then defined as the percentage contribution of each factor to the total theoretical maximum effect summed over all factors. To establish between-group significance of the difference in importance weights, we computed the standard error of the difference based on the standard errors of the weights and subsequently conducted standard *t*-tests. Between-group difference in the frequency to advise surgery or comfort care was assessed by the Chi^2^-test. We divided the participant—reported work experience subgroups in: less experienced (0–5 years), moderately experienced (5–15 years) and highly experienced (>15 years) physicians.

## Results

We invited 109 neonatal care experts, including 88 neonatologists and 21 pediatric surgeons. The choice experiment was completed by 45 neonatologists, 16 pediatric surgeons and one neonatology physician assistant. This amounted to a total of 62 participants out of 109 invitees (response rate 57%) (Table 2). Fourteen (23%) participants were excluded from subgroup analyses because they opted to omit occupational information: three from the specialty subanalysis, six from the center subanalysis and five from the work experience subanalysis.

**Table 2:** Participants per center.

Center	Neonatologists	Pediatric surgeons	Totals
A	12	6	18
B	12	4	16
C	6	2	8
D	7^a^	-	7
E	3	-	3
F	3	-	3
G	1	2	3
H	2	-	2
I	-	2	2
Total	45	16	62

^a^ Including one neonatology physician assistant (i.e., especially trained registered nurse).

### Nationwide results

Factor weights for nationwide choice analysis and for the subgroup of neonatologists and pediatric surgeons are displayed in Table 3. Model fit of the estimated nationwide model was calculated at a McFadden’s *ρ*^2^ value of 0.27. The mean absolute deviation was 5.8%, indicating that on average the predicted probability (percentage) of a recommendation to operate by the model was 5.8% higher or lower than the observed percentage of experts choosing for operation in the choice experiment. Out of the fourteen factors, eleven had significant impact on the decision. *Sex* (*p* = 0.14), *growth since birth* (*p* = 0.25) and *estimated parental capacities* (*p* = 0.06) did not significantly affect the decision in the nationwide model.

**Table 3:** Estimated linear weight (beta coefficient) of each factor per level, of the nationwide analysis and the neonatologist and pediatric surgeon subgroups.

Factor	Nationwide (*N* = 62 participants) coefficient (*p*-value) *OR [95% CI]*	Neonatologists (*N* = 44 participants) coefficient (*p*-value) *OR [95% CI]*	Pediatric surgeons (*N* = 14 participants) coefficient (*p*-value) *OR [95% CI]*	Difference subgroups (Neo vs. PS) *p*-value
Sex	0.17 (0.14) 1.18 [0.95–1.47]	0.21 (0.17) 1.23 [0.95–1.60]	0.27 (0.32) 1.31 [0.77–2.21]	0.84
Gestational age	0.45 (<0.001^**^) 1.56 [1.38–1.77]	0.46 (<0.001^**^) 1.59 [1.37–1.84]	0.45 (0.006^*^) 1.57 [1.14–2.17]	0.97
Birth weight	0.58 (<0.001^**^) 1.78 [1.56–2.04]	0.60 (<0.001^**^) 1.81 [1.55–2.12]	0.57 (0.005^*^) 1.76 [1.28–2.42]	0.87
Perinatal asphyxia	0.32 (<0.001^**^) 1.37 [1.20–1.57]	0.39 (<0.001^**^) 1.47 [1.26–1.72]	0.16 (0.34) 1.17 [0.85–1.61]	0.21
Congenital comorbidity	0.69 (<0.001^**^) 1.99 [1.71–2.33]	0.68 (<0.001^**^) 1.97 [1.64–2.36]	0.90 (<0.001^**^) 2.47 [1.70–3.58]	0.29
Course pre-NEC	0.30 (<0.001^**^) 1.35 [1.15–1.58]	0.28 (0.003^*^) 1.33 [1.10–1.60]	0.51 (0.014^*^) 1.66 [1.11–2.48]	0.33
Postnatal age	0.22 (0.005^*^) 1.24 [1.07–1.44]	0.28 (0.002^*^) 1.32 [1.11–1.58]	0.04 (0.82) 1.04 [0.73–1.48]	0.24
Growth since birth	0.08 (0.25) 1.09 [0.94–1.25]	0.01 (0.87) 1.01 [0.86–1.20]	0.36 (0.03^*^) 1.44 [1.03–2.01]	0.07
Cerebral ultrasound	1.40 (<0.001^**^) 4.06 [3.39–4.86]	1.43 (<0.001^**^) 4.19 [3.40–5.16]	1.59 (<0.001^**^) 4.92 [3.18–7.63]	0.52
Lung function	0.36 (<0.001^**^) 1.43 [1.23–1.67]	0.40 (<0.001^**^) 1.49 [1.25–1.79]	0.29 (0.10) 1.33 [0.94–1.89]	0.57
Hemodynamics	0.76 (<0.001^**^) 2.15 [1.85–2.49]	0.67 (<0.001^**^) 1.96 [1.65–2.32]	1.29 (<0.001^**^) 3.63 [2.41–5.46]	0.008^*^
Cerebral oxygenation	0.31 (<0.001^**^) 1.36 [1.17–1.59]	0.33 (<0.001^**^) 1.39 [1.16–1.66]	0.47 (0.02^*^) 1.60 [1.09–2.37]	0.51
Parental preferences	0.65 (<0.001^**^) 1.91 [1.66–2.19]	0.62 (<0.001^**^) 1.86 [1.58–2.20]	0.81 (<0.001^**^) 2.24 [1.64–3.05]	0.31
Est. parental capacities	0.15 (0.06) 1.16 [1.00–1.35]	0.13 (0.13) 1.14 [0.96–1.36]	0.27 (0.16) 1.31 [0.90–1.92]	0.52
Constant	−6.18 (<0.001^**^)	−6.39 (<0.001^**^)	−7.28 (<0.001^**^)	
Model fit	*ρ*^2^ = 0.27	*ρ*^2^ = 0.26	*ρ*^2^ = 0.37	
**Observed advices:**				
Comfort care	*n* = 882 (41%)	*n* = 662 (43%)	*n* = 184 (38%)	0.03^*^
Surgery	*n* = 1,288 (59%)	*n* = 878 (57%)	*n* = 306 (62%)	

Model: binary logistic regression. Decision: recommendation to operate (1) or not (0). Beta coefficient, odds ratio and significance (null model vs. estimated model) is provided for each factor. Recommendation displayed as the number of answers (%) provided per group. *p*-value of difference in recommendation is based on Chi^2^-analysis, *p*-value of between-group difference in factor impact is based on a *t*-test. Subgroup numbers do not add up to the total of the nationwide model due to four patients that omitted occupational information. Neo, neonatologists; PS, pediatric surgeons; NEC, necrotizing enterocolitis; NIRS, near-infrared spectroscopy; Est., estimated; OR, odds ratio (indicating the odds of a recommendation to operate with one level increase of a factor); CI, confidence interval.

* *p* < 0.05.

** *p* < 0.001.

**Figure 3:**
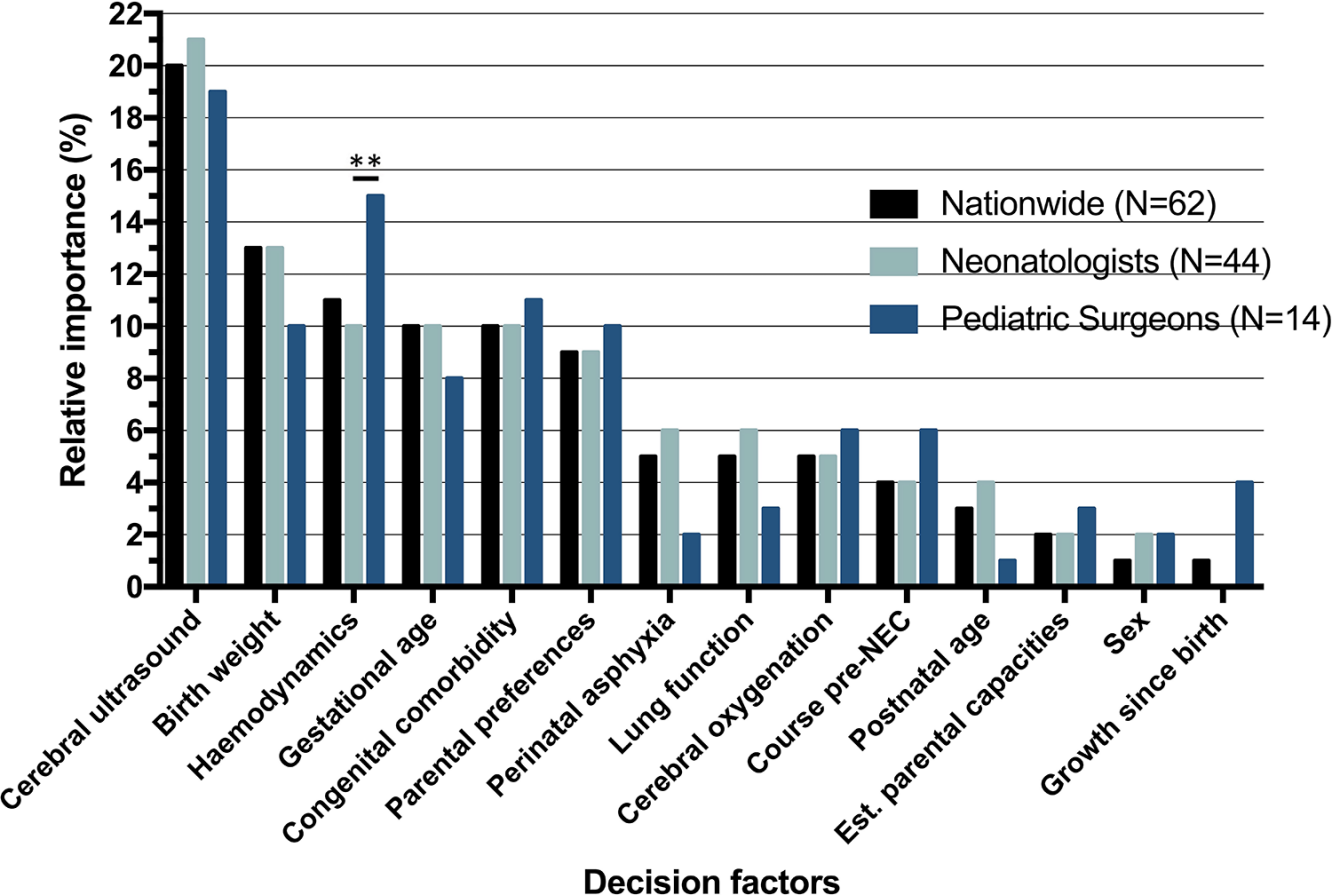
Relative importance (RI) of decision factors, nationwide and per specialty. *RI determined by*: $\frac{\mathrm{beta}\;\mathrm{coefficient}\;\times \;\mathrm{factor}\;\mathrm{range}}{\mathrm{SUM}\;(\mathrm{all}\;\mathrm{beta}\;\mathrm{coefficients}\;\times \;\mathrm{all}\;\mathrm{factor}\;\mathrm{ranges})}$. **Significant difference (*p* < 0.01) based on standard error of the difference between beta coefficients.

Relatively, *cerebral ultrasound* [OR = 4.06, 95% confidence interval (CI) = 3.39–4.86] had the largest impact on the critical decision (Table 3). The most impactful (RI equal to or higher than 10%) and statistically significant factors were *cerebral ultrasound* (RI = 20%), *birth weight* (RI = 13%), *hemodynamics* (RI = 11%), *gestational age* (RI = 10%), *congenital comorbidity* (RI = 10%) (Figure 3). Having discussed the RI of individual factors, the probability of the decision to operate is the combination of all decision factors included in the choice analysis (Figure 2).

### Neonatologists or pediatric surgeons

The McFadden’s *ρ*^2^ of the neonatologist group (*n* = 44) and the pediatric surgeon (*n* = 14) group were 0.26 and 0.37, respectively. Table 3 depicts choices and regression analysis outcomes of neonatologists and pediatric surgeons. Overall, pediatric surgeons were somewhat more inclined to advice surgery when compared to neonatologists (62% vs. 57% of scenario answers, *p* = 0.03). *Cerebral ultrasound* had the greatest RI for both neonatologists and pediatric surgeons (RI 21%, and 19% respectively, *p*-value of the difference: 0.52). *Hemodynamics* was the sole factor that differed significantly in impact on the decision between the two professions [RI 10% (neonatologists) vs. 15% (pediatric surgeons), *p* = 0.008].

### Between-center differences

**Figure 4:**
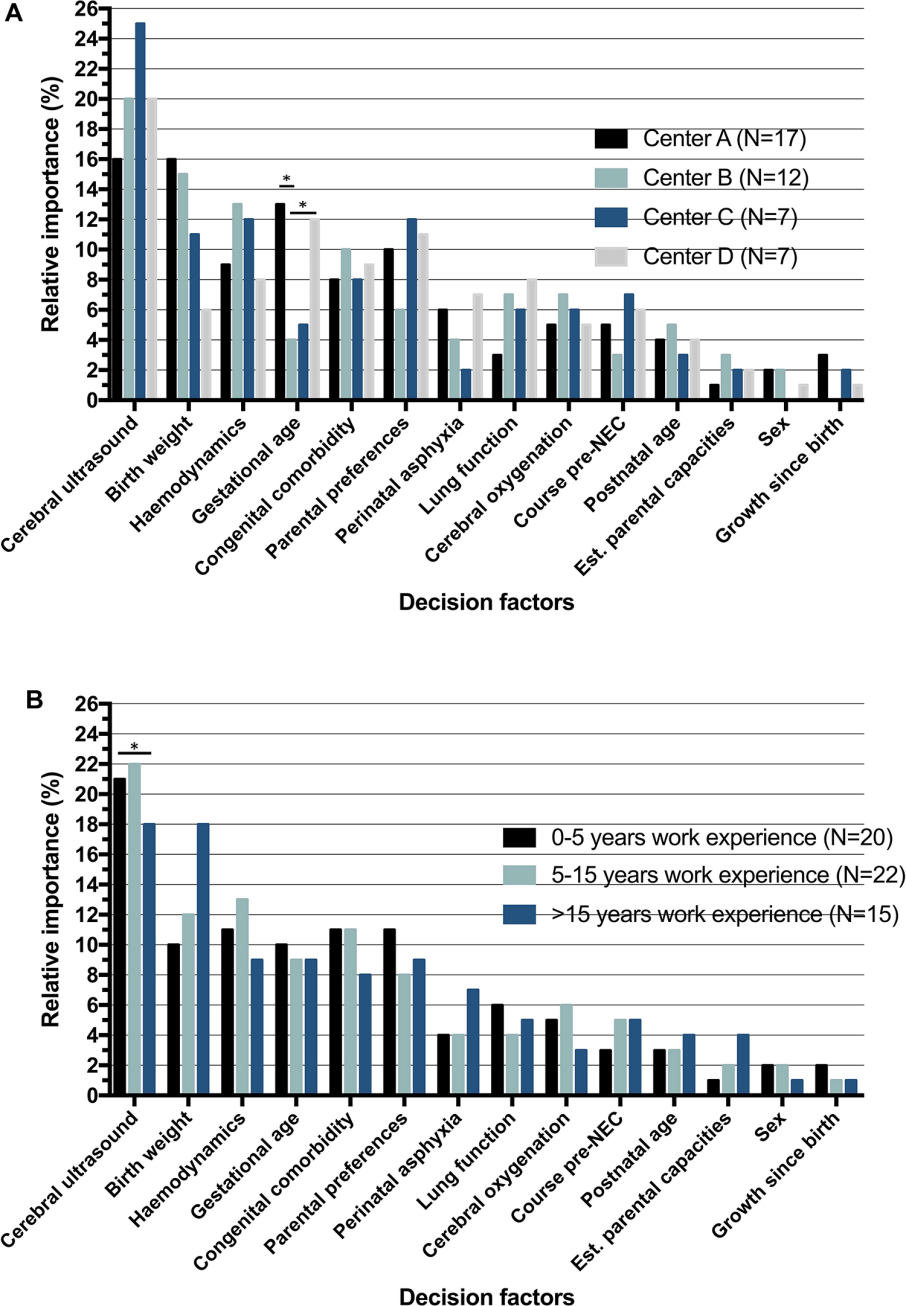
Relative importance (RI) of decision factors, (**A**) per center and (**B**) per work experience. *RI determined by*: $\frac{\mathrm{beta}\;\mathrm{coefficient}\;x\;\mathrm{factor}\;\mathrm{range}}{\mathrm{SUM}\;(\mathrm{all}\;\mathrm{beta}\;\mathrm{coefficients}\;\times \;\mathrm{all}\;\mathrm{factor}\;\mathrm{ranges})}$. *Significant difference (*p* < 0.05) based on standard error of the difference between beta coefficients.

RI of factors is displayed in Figure 4 for the four neonatal care centers with the most participants: center A (*n* = 17), center B (*n* = 12), center C (*n* = 7) and center D (*n* = 7). Other centers had participant numbers of <7 for subanalysis, resulting in limited value for additional analyses. The frequency of recommendation for surgery varied significantly between centers with 53% in center A, 63% in center B, 58% in center C and 57% in center D (*p* = 0.03). Interpretation of *cerebral ultrasound* was the factor with most impact on the decision in all four centers. In the estimated model for center A, *birth weight* (RI = 16%) had an equal impact on the decision as *cerebral ultrasound* (RI = 16%). Four factors consistently had a significant effect in per-center regression analysis of all centers, including: *cerebral ultrasound*, *hemodynamics*, *congenital comorbidity* and *parental preferences*. Factors with no significant impact on the decision in all four centers were *sex*, *growth since birth* and *estimated parental capacities*.

### Work experience

Subgroup analysis for years of working experience as a specialist is displayed in Table 4. The overall trend for all groups was more scenario answers in favor of surgery, with 398 (57%) answers in the 0–5 years, 476 (62%) answers in the 5–15 years and 289 (55%) answers in the >15 years work experience group. Comparing the linear weight of decision factors between the groups, there was only a significant difference in *cerebral ultrasound* between 5 and 15 and >15 years experience groups (beta coefficient 1.72 vs. 1.15, *p* = 0.02). In per-group analyses, *course pre-NEC* was not a significant factor in the decision for the 0–5 years work experience group (*p* = 0.09), whereas it was for the other two groups (5–15 years *p* = 0.02; >15 years *p* = 0.047). Conversely, *cerebral oxygenation* was not significant in the >15 years group (*p* = 0.18), while in the 0–5 years experience (*p* = 0.02) and 5–15 years experience (*p* = 0.001) groups it was.

**Table 4:** Estimated linear weight of the work experience subgroup analysis, divided in three groups: 0–5 years (group 1), 5–15 years (group 2) and >15 years (group 3) of working experience as a neonatologist or pediatric surgeon.

Factor	0–5 years (1) (*N* = 20 participants) coeff. (*p*-value)	5–15 years (2) (*N* = 22 participants) coeff. (*p*-value)	>15 years (3) (*N* = 15 participants) coeff. (*p*-value)	Difference (1) vs. (2) *p*-value	Difference (1) vs. (3) *p*-value	Difference (2) vs. (3) *p*-value
Sex	0.28 (0.18)	0.25 (0.22)	0.10 (0.65)	0.92	0.57	0.63
Gestational age	0.47 (<0.001^**^)	0.46 (<0.001^**^)	0.39 (0.002^*^)	0.97	0.66	0.69
Birth weight	0.51 (<0.001^**^)	0.63 (<0.001^**^)	0.78 (0.006^*^)	0.48	0.14	0.44
Perinatal asphyxia	0.33 (0.009^*^)	0.28 (0.02^*^)	0.44 (0.001^**^)	0.79	0.55	0.38
Congenital comorbidity	0.80 (<0.001^**^)	0.83 (<0.001^**^)	0.51 (<0.001^**^)	0.85	0.19	0.14
Course pre-NEC	0.25 (0.09)	0.35 (0.02^*^)	0.31 (0.047^*^)	0.61	0.77	0.84
Postnatal age	0.22 (0.11)	0.21 (0.11)	0.29 (0.07)	0.98	0.75	0.73
Growth since birth	0.17 (0.19)	0.09 (0.48)	−0.04 (0.78)	0.67	0.28	0.50
Cerebral ultrasound	1.53 (<0.001^**^)	1.72 (<0.001^**^)	1.15 (0.004^*^)	0.42	0.13	0.02^*^
Lung function	0.44 (0.002^*^)	0.31 (0.03^*^)	0.34 (0.03^*^)	0.49	0.62	0.88
Hemodynamics	0.77 (<0.001^**^)	0.99 (0.01^*^)	0.62 (<0.001^**^)	0.25	0.46	0.07
Cerebral oxygenation	0.34 (0.02^*^)	0.45 (0.001^*^)	0.21 (0.18)	0.57	0.53	0.24
Parental preferences	0.80 (<0.001^**^)	0.60 (<0.001^**^)	0.60 (<0.001^**^)	0.28	0.30	0.97
Est. parental capacities	0.05 (0.70)	0.15 (0.29)	0.24 (0.10)	0.63	0.36	0.65
Constant	−6.76 (<0.001^**^)	−6.85 (<0.001^**^)	−6.05 (0.07)			
Model fit	*ρ*^2^ = 0.30	*ρ*^2^ = 0.33	*ρ*^2^ = 0.23			
**Observed advices:**						
Comfort care	*n* = 302 (43%)	*n* = 294 (38%)	*n* = 236 (45%)	0.05	0.05	0.01^*^
Surgery	*n* = 398 (57%)	*n* = 476 (62%)	*n* = 289 (55%)			

Model: binary logistic regression. Decision: recommendation to operate (1) or not (0). Beta coefficient and significance (null model vs. estimated model) is provided for each factor. Recommendation displayed as the number of answers (%) provided per group. *p*-value of difference in recommendation is based on Chi^2^-analysis, *p*-value of between-group difference in factor impact is based on a *t*-test. Neo, neonatologists; PS, pediatric surgeons; NEC, necrotizing enterocolitis; NIRS, near-infrared spectroscopy; Est., estimated; Coeff., coefficient.

* *p* < 0.05.

** *p* < 0.001.

## Discussion

In this study, we applied choice analysis techniques to identify, analyze and codify the weight of factors in the decision for comfort care or surgery in a critically ill neonate with surgical NEC. BAIT demonstrated that, both over the nationwide analysis and subanalyses, *cerebral ultrasound* was the factor with most impact on the decision. Notably, *birth weight* and *gestational age* were the second and third most important decision factors for neonatologists, whereas for pediatric surgeons these were *hemodynamics* and *congenital comorbidity*. Factors of significant impact in all centers were *cerebral ultrasound*, *congenital comorbidity*, *hemodynamics* and *parental preferences* and the maximum difference in number of recommendations for surgery between centers was 10 percentage points. We attained a choice model, equipped with the decision factor weights, with a mean absolute deviation of 5.8%.

Interpreted *Cerebral ultrasound* prognosis was the decision factor with most impact, in the nationwide model and all other subgroup models. Long-term neurodevelopmental impairment, including motor deficits, sensory deficits, behavioral issues and cognitive impairment, is a well-established association in infants suffering from both medical and surgical NEC (21–23). Meta-analysis has also established that infants surgically treated for NEC are even at a 16% higher risk for neurodevelopmental impairment (23). Brain injury visualized on cerebral ultrasonography is associated with neurodevelopmental delay (24, 25). Similarly, low birth weight and gestational age are well-known predictors of long-term neurodevelopmental impairment and had large RI in our study (26). Hence, the dominant weight of *cerebral ultrasound* and also the large impact of *birth weight* and *gestational age* may reflect the perceived importance of long-term neurodevelopmental function, i.e., the recommendation for NEC surgery becomes much less desirable for participants due to the long-term cognitive and functional prognosis after surgery.

In accordance with a recent study from the USA (27), we observed a significant difference between Dutch neonatologists (57% of cases) and pediatric surgeons (62% of cases) in recommending surgery. An explanation for a less pronounced difference in surgery recommendations in our study, may be the differences in the health care and insurance systems (28, 29). Factors with the second and third highest RI varied between neonatologists and pediatric surgeons. These findings are in accordance with two potentially different thought-processes between the two specialist groups: (1) neonatologists leaning more towards the consideration whether the child will be majorly impaired in the long-term (*birth weight*, *gestational age*) and; (2) pediatric surgeons leaning more towards the consideration whether the child may or may not survive NEC surgery (*hemodynamics*, *congenital comorbidity*). This is also in accordance with the recent USA survey study mentioned earlier (27).

Despite mainly congruent results in work experience subanalysis, *course pre-NEC* was not significant in the 0–5 years experience group (*p* = 0.09) and *cerebral oxygenation* was not significant in the >15 years experience group (*p* = 0.18). Association between low cerebral oxygenation (NIRS, near infrared spectroscopy) and NEC development, survival after NEC surgery and neurodevelopmental impairment has been found (10, 30–32). In the perspective of work experience, the findings possibly simulate a transition between the less and more experienced specialists: younger specialists focus more on more recently implemented monitor parameters (cerebral oxygenation), whereas more experienced specialists rely more on their clinical view (clinical course prior to NEC). As NIRS is not used in all centers, this relative lack of experience with this technique might also have influenced results due to certain participants blunting its relevance in the decision.

Between centers we noted a maximum difference of 10 percentage points in the frequency of surgery recommendation and a maximum difference of 9 percentage points between the RI of *cerebral ultrasound* between centers. Nevertheless, looking at the relatively small groups in per-center analyses, these differences could probably be explained by a single or a few participants, challenging their clinical significance and accentuating the relative intrinsic complexity of NEC decision making. Out of the total of fourteen factors, four were consistently significant and three consistently insignificant factors across the different center subgroups. This highlights the consonance of decision making in critically ill neonates within each center. The Dutch guidelines command that continuation of treatment for a critically ill neonate is conditional, tailored to the specific case (33). Our study reassuringly shows that, despite subtle differences, Dutch experts in (neonatal) health care settings have a relatively harmonious set of medical expertise, norms and values, to be tailored to a medical decision in a child’s best interest.

Non-significant factors nationwide were *sex*, *growth since birth* and *estimated parental capacities*. *Sex* was included as a factor based on evidence that extremely preterm females perform better in overall mortality and morbidity rates compared to males (34–37). Yet, an elaborate meta-analysis found no significant impact of sex on cognitive outcome of extreme/very preterm infants (38). Our study confirms that *sex* does not project significantly in decision making for surgical NEC. *Growth since birth* may be non-significant due to its multifactorial nature and the relatively early onset of NEC itself. The factor *estimated parental capacities* was not significant. Conversely, *parental preferences* was, which confirms that clinicians do find parental involvement important. Engagement of parents in NEC care and decisions also improves parental satisfaction (39). Hence, our study reflects that the desire of parents for treatment more accurately represents the perspectives for future care of an infant than our subjective estimation of parental capacities.

The BAIT decision-analysis tool enabled us to capture the expertise of a nationwide panel of neonatal and pediatric surgical specialists. It clearly provided insights into factors affecting their choices regarding one of the most difficult decisions in neonatology. A limitation is that captured factors are considered important by Dutch physicians and may not be generalizable, considering different ethical circumstances and attitudes surrounding the decision may exist worldwide. Moreover, it should be considered that our technique provides decision transparency but does not dictate which choice is “the best” in this nuanced critical decision. Nevertheless, this methodology can be applied to identify decision factor weights internationally or for parents. In the future, a more elaborate European or worldwide choice experiment could offer insight into factors that influence this decision in other countries, thereby shedding a light on the possible differences between countries and cultures.

Limited numbers of inclusions in the neonatal center subgroups did not allow for elaborate between-center comparison. In the nationwide model, as well as in the group of pediatric surgeons there was a relative overrepresentation of the UMCG. This might be due to the fact that the UMCG, a Center of Expertise for NEC as appointed by the Dutch Ministry of Health, initiated the study so that UMCG staff was more inclined to complete the study. Also, the 14 clinical variables and their ranges were determined by a UMCG expert group, which may have limited generalizability. However, given the nature of the experiment, potentially irrelevant variables added by the UMCG expert group would turn out to be insignificant in the overall study results, based on the input of all participants. Another limitation was that our choice experiment did not consist of real-life cases, but of hypothetical, computer-generated scenarios completed in a software environment. Hence, validation with real-life cases is required prior to its potential application in medical practice as a dynamic decision transparency tool. As the model can be updated based on encountered real-life cases, it will continuously progress alongside advances in neonatal and pediatric surgical care, harnessing the power of artificial intelligence (2).

## Conclusion

As shown in this study, choice analysis may be utilized to educate us about the weight of factors in medical decision making and reflect upon them. Our methodology exposed between-group differences and showed that, despite some variation, Dutch neonatal care experts have a generally harmonious set of medical expertise, norms and values in critical decision making in surgical NEC. After validation, the model may serve as a decision aid for neonatologists, pediatric surgeons and parents of patients.

## Data Availability

The raw data supporting the conclusions of this article will be made available by the authors, without undue reservation.
